# Supplementary material for: Development of a computer-aided design software for the quantitative evaluation of aesthetic damage
Source: PLoS One. 2019 Dec 18;14(12):e0226322. doi: 10.1371/journal.pone.0226322 (PMC6919621; doi:10.1371/journal.pone.0226322)
Supplement: S1 Code site — 1. (ZIP) [file pone.0226322.s001.zip › [EESC_jr][Projeto]Relatorio_07_21/vendors/select2/docs/_includes/options/events/jquery.html]

## Public jQuery events

### What events will Select2 trigger?

Select2 will trigger a few different events when different actions are taken using the component, allowing you to add custom hooks and perform actions.

change
:   Triggered whenever an option is selected or removed.

select2:close
:   Triggered whenever the dropdown is closed.

select2:closing
:   Triggered before the dropdown is closed. This event can be prevented.

select2:open
:   Triggered whenever the dropdown is opened.

select2:opening
:   Triggered before the dropdown is opened. This event can be prevented.

select2:select
:   Triggered whenever a result is selected.

select2:selecting
:   Triggered before a result is selected. This event can be prevented.

select2:unselect
:   Triggered whenever a selection is removed.

select2:unselecting
:   Triggered before a selection is removed. This event can be prevented.

### Does Select2 include extra information in these events?

{% include options/not-written.html %}

### How can I attach listeners for these events?

{% highlight js linenos %}
$('select').on('select2:select', function (evt) {
// Do something
});
{% endhighlight %}
{% include options/not-written.html %}

### What events does Select2 listen for?

Select2 will listen for the `change` event on the
`<select>` that it is attached to. If you make any
external changes that need to be reflected in Select2 (such as changing the
value), you should trigger this event.

{% highlight js linenos %}
$('select').val('US'); // Select the option with a value of 'US'
$('select').trigger('change'); // Notify any JS components that the value changed
{% endhighlight %}

### Can I trigger an event other than `change` to notify Select2 of changes?

It's common for other components to be listening to the `change`
event, or for custom event handlers to be attached that may have side
effects. Select2 does not have a custom event (like
`select2:update`) that can be triggered other than
`change`. You can rely on jQuery's event namespacing to limit
the scope to Select2 though by triggering the `change.select2`
event.

{% highlight js linenos %}
$('select').val('US'); // Change the value or make some change to the internal state
$('select').trigger('change.select2'); // Notify only Select2 of changes
{% endhighlight %}

### What events can be prevented? How can I prevent a selection from being made?

{% include options/not-written.html %}
